# An 8-year clinical experience with diagnosis and treatment of adrenal lesions with calcification

**DOI:** 10.1038/s41598-022-10110-5

**Published:** 2022-04-12

**Authors:** Jun Dai, Jialing Xie, Kai Yang, Wei He, Fukang Sun, Danfeng Xu, Min Jiang, Juping Zhao

**Affiliations:** 1 Department of Urology, Ruijin Hospital, Shanghai Jiao Tong University School of Medicine, Shanghai, 200025 China; 2 Department of Pathology, Ruijin Hospital, Shanghai Jiao Tong University School of Medicine, Shanghai, 200025 China; 3 Department of Orthopaedics, Shanghai Key Laboratory for Prevention and Treatment of Bone and Joint Diseases, Shanghai Institute of Traumatology and Orthopaedics, Ruijin Hospital, Shanghai Jiao Tong University School of Medicine, 197 Ruijin 2nd Road, Shanghai, 200025 China

**Keywords:** Endocrinology, Urology

## Abstract

Adrenal lesions with calcification are uncommon and surgical indication remains controversial. We evaluate rational indications for surgical intervention of adrenal lesions with calcification. From 2013 to 2021, 75 adrenal lesions with calcification managed with surgery had necessary studies for evaluation of rational surgical indication. Clinical benefit was defined as relief of symptoms or/and removal of the malignant tumors. Influencing factors for clinical benefit were evaluated by logistic regression. During the past 8-year period, 5057 patients received adrenal surgery in our center and 75 (1.5%) patients were accompanied with calcification, including 34 males and 41 females with a median age of 54 years (IQR = 41–63 years). The median maximum diameter of calcified adrenal lesions on preoperative CT imaging was 4.2 cm (IQR = 3.0–5.9 cm). Clinical benefit was achieved in 22 cases, including 4 cases of malignant tumors and 18 cases of relieved clinical symptoms. Correlation analysis indicated that maximum diameter of the lesion was significantly correlated with clinical benefit (*p* = 0.025). The maximum diameter in benefit group *vs.* non-benefit group was 5.5 cm (IQR = 3.7–7.4 cm) *vs.* 3.7 cm (IQR = 2.8–5.4 cm). AUC of the maximum diameter ROC curve of adrenal lesions was 0.662. The diameter, sensitivity and specificity corresponding to the maximum Youden index value were 4.5 cm, 0.682 and 0.623, respectively. Clinical benefit was not significantly correlated with calcification distribution (peripheral or internally scattered) (*P* = 0.106), calcification area ≥ 50% (*P* = 0.617) and internal enhancement of the lesion (*P* = 0.720). Adrenal lesions with calcification are mostly benign. Clinical benefit is significantly correlated with the maximum diameter of the lesion and 4.5 cm may be considered as the cutoff point of surgical intervention.

## Introduction

Most adrenal lesions with calcification are pathologically benign and the clinical benefit is significantly correlated with the maximum diameter of the lesion. Strict control of the surgical indication can avoid unnecessary surgical intervention.

The adrenal gland plays an important role in regulating physiological functions. It is easily affected by various diseases. Although more cases of adrenal calcification have been detected with the extensive application of computed tomography (CT) and other imaging techniques, they are still uncommon^1,2^ and therefore there is limited experience about the clinical diagnosis and treatment of adrenal lesions with calcification. In addition, whether surgery is necessary for such lesions remains controversial. In clinical practice, relief of symptoms and removal of the malignant tumor are clinical expected commonly as “clinical benefit”. In this study, we make a retrospective analysis of the clinical data of patients with adrenal diseases who received surgical treatment in our high-volume adrenal disease center during the past 8 years and discuss the rational indications for surgical treatment of adrenal lesions with calcification.

## Results

### Clinical manifestation and laboratory findings

Altogether 5057 patients with adrenal diseases received surgical treatment during the past 8-year period, of whom 75(1.5%) were found to have calcification, involving 34 males and 41 females with a median age of 54 years (IQR = 41–63 years). The lesions were located on the left side in 33(44%) cases and right side in 42(56%) cases (Table 1).

**Table 1:** The demographics and clinical parameters of the patients with calcified adrenal lesion.

Parameter		No. (%)
Gender	Male	34 (45%)
	Female	41 (55%)
Age (years)	54 (IQR = 41–63)	
Side	Left	33 (44%)
	Right	42 (56%)
Maximum diameter of adrenal lesion on CT (cm)	4.2 (IQR = 3.0–5.9)	
Clinical symptoms	No	39 (52.0%)
	Hypertension	26 (34.7%)
	Flank/back pain	8 (10.7%)
	Lower limb edema	1 (1.3%)
	Complicated pneumonia	1 (1.3%)
Preoperative endocrinology evaluation	Non-function	71 (94.7%)
	Hyperaldosteronism	3 (4%)
	Cushing syndrome	1 (1.3%)
Calcification location	Periphery	57 (76%)
	Scattered inside	18 (24%)
Calcification area > 50% of the adrenal lesion	No	66 (88%)
	Yes	9 (12%)
Enhancement on CT	No	50 (66.7%)
	Yes	25 (33.3%)
Surgical approach	Retroperitoneoscopy	39 (52%)
	Transperitoneal laparoscopy	27 (36%)
	Open	9 (12%)
Preservation of normal adrenal gland	Not preserved	38 (50.7%)
	Preserved	37 (49.3%)
Follow-up (mon)		36.5 (IQR = 25.5–63.0)

No. = number; CT = computed tomography; IQR = interquartile range.

Of the 75 patients with calcified adrenal lesions, 39(52.0%) cases were accidently discovered, 26(34.7%) had preoperative hypertension, 8(10.7%) had self-perceived low back pain, one(1.3%) had lower extremity edema, and one(1.3%) had complicated pulmonary infection (Table 1). After adrenectomy, hypertension was improved in 12(46.2%) of the 26 hypertensive patients; low back pain was relieved in 8(75%) patients whose tumor median size was 5.8 cm (IQR = 4.0–10.8 cm); lower extremity edema was relieved in one patient. After controlling the complicated pulmonary infection, surgery was performed in this patient. Of the 75 patients with calcified adrenal lesions, endocrine function test showed no function in 71(94.7%), primary hyperaldosteronism in 3(4%), and Cushing syndrome in one(1.3%).

### Characteristics on CT imaging

The median value of maximum diameter of the calcified adrenal lesions was 4.2 cm (IQR = 3.0–5.9 cm) as measured by preoperative CT. Of the 75 adrenal lesions, the calcification located in the periphery in 57(76%) cases, and scattered inside in the reaming 18 cases. The calcification area involved less than 50% of the adrenal lesion in 66(88%) cases, and ≥ 50% in the remaining 9(12%) cases. Of them, 9 were all benign lesions, including 2 cysts, 1 pseudocyst, 2 myeloid lipomas, 2 angiomas, 1 schwannoma, and 1 ganglioneuroma and the median tumor diameter was 4 cm (IQR = 3.1–4.7 cm). Contrast-enhanced CT scan showed no-enhancement in 50(66.7%) cases and partial enhancement in the remaining 25 cases. The individual characteristics of CT for the 14 sub-groups were listed in Table 2 respectively.

**Table 2:** CT imaging and pathology of adrenal lesion accompanied with calcification.

Type	No	%	Characteristics
Adrenal cyst	29	38.7	Calcification is usually located in the periphery of the cyst with water-like uniform density inside with no enhancement on CT imaging. Microscopically, the fibrous capsule wall tissue is covered with the pseudostratified ciliated columnar epithelium. Most adrenal cysts are hemorrhagic, with hemosiderosis and calcification contained in the cyst wall
Adrenal pseudocyst	2	2.7	Calcification was located peripherally on CT imaging. Microscopically, the wall is made up of inflammatory granulation tissue or fibrous tissue with no coverage of epithelial cells on the cyst wall surface
Adrenal myelolipoma	10	13.3	Fat density can be seen on the CT image. Microscopically, fat lipocytes in myelolipoma are uniformly distributed, with scattered calcifications inside but the CT value of calcification is not high
Adrenal mature teratoma	2	2.7	Calcification lesions are mostly patchy or curved, with the CT value high as 500HU. Histologically, well differentiation tissues contain 3 germ cell layers (ectoderm, mesoderm and endoderm)
Adrenocortical adenoma	10	13.3	Calcification is scattered and can be seen in the periphery or inside. Microscopically, it is composed of clear and compact cells; the tumor cells are arranged in a cord-like or acinar manner, with mitosis rarely seen
Adrenal angiolymphangioma	6	8.0	Calcification located in the periphery and presented a patchy shape on the CT image. In some cases, partitioned calcification foci can be seen inside. Histologically, the cystic spaces were lined by a single layer of flattened or focally hobnailed cells without atypia resembling that of normal lymphatics
Adrenal schwannoma	5	6.7	Calcification mainly located in the periphery on the CT image, forming an “egg shell-like” shape encircling the tumor. Microscopically, the tumor cells are spindle-shaped with no clear boundaries between cells, and cells are arranged in a whirlpool or palisade manner
Adrenal pheochromocytoma	1	1.3	Calcification presented a ring-like shape on the CT image, with a CT value larger than 500HU. Microscopically, most tumor cells are polygonal with rich granular or vacuolar alkalophilic cytoplasm and clear nucleoli, and cells are arranged in a nest-like, trabecular or acinar shape, and strongly positive Chromogranin A in immunohistochemistry
Adrenal hematoma	3	4.0	Calcification is mainly distributed in the peripheral wall of the hematoma and partitioned inside in a multiple manner. Microscopically, hemorrhage is localized within the adrenal tissue, with normal adrenal tissue surrounding the calcification
Ganglioneuroma	2	2.7	Calcification in ganglioneuroma presents as nodules inside the tumor on the CT image. Microscopically, patchy or scattered maturely differentiated gangliocytes are seen in unmyelinated nerve fibers
Adrenal macronodular hyperplasia	1	1.3	Calcification presents as a single spot inside the lesion on CT image. Microscopically, the adrenal cortex was occupied by multiple nodular lesions composed mostly of clear cells. In the internodular regions, no evidence of cortical architecture was observed
Adrenal cortical carcinoma	2	2.7	Calcification is spotty on CT image, scattering inside the tumor with a relatively low CT value of calcification (< 100Hu). The maximum diameter is relatively large, and the rumor is lobulated with an unclear boundary. Microscopically, most cells are dark, with a patchy or focal density showing nuclear abnormalities, clear nucleoli and nuclear mitosis, invading blood vessels or penetrating the capsule with extensive hemorrhage and necrosis
Liposarcoma	1	1.3	Calcification presents as a sporadic and punctate distribution inside the tumor on the CT image, with an CT value of calcification less than 100 HU. Microscopically, adipoblasts and mature adipocytes in different differentiation stages show obvious heterogeneity
Gastric adenocarcinoma metastatic to the adrenal gland	1	1.3	Calcification presents a scattered spotty distribution on the CT image, with a CT value of calcification lower than 100 HU. Microscopically, there is little lipid, and typical gastric adenocarcinoma cells

### Surgical approach and pathology

Retroperitoneoscopy was performed in 39(52%) cases, transperitoneal laparoscopy in 27(36%) cases, and open surgery in 9(12%) cases. The median value of maximum diameter in the 9 cases of open surgery was 11.8 cm (IQR = 7.5–13.7 cm). The normal adrenal gland was not preserved in 38(50.7%) cases, including 4 cases of malignant tumors. The median maximum diameter of the remaining 37(49.3%) cases with the adrenal gland preserved was 4.1 cm(IQR = 2.9–5.3 cm), and all of them were pathologically benign.

**Figure 1:**
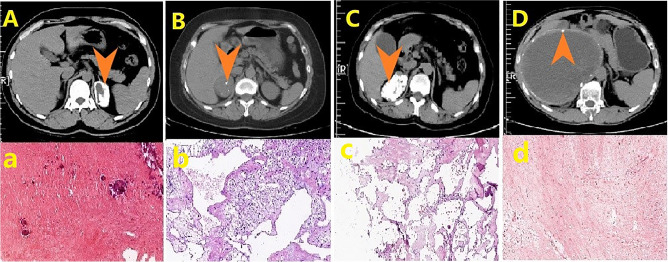
**(A)** Computed tomography (CT) of adrenal cyst: peripheral calcification with water-like uniform density inside with no enhancement. **(a)** hematoxylin and eosin (H&E) staining of adrenal cyst, magnification × 100: fibrous capsule wall tissue is covered with the pseudostratified ciliated columnar epithelium. **(B)** CT of adrenal cortical adenoma: scattered calcification inside. **(b)** H&E staining of adrenal cortical adenoma, magnification × 100: composed of clear and compact cells, the tumor cells are arranged in a cord-like manner. **(C)** CT of benign lymphangioma: peripheral calcification with patchy shape. **(c)** H&E staining of benign lymphangioma, magnification × 100: cystic spaces were lined by a single layer of flattened hobnailed cells without atypia. **(D)** CT of adrenal schwannoma: peripheral calcification with an “egg shell-like” shape encircling the tumor. **(d)** H&E staining of adrenal schwannoma, magnification × 100: tumor cells are spindle-shaped with no clear boundaries between cells, and cells are arranged in a whirlpool manner.

**Figure 2:**
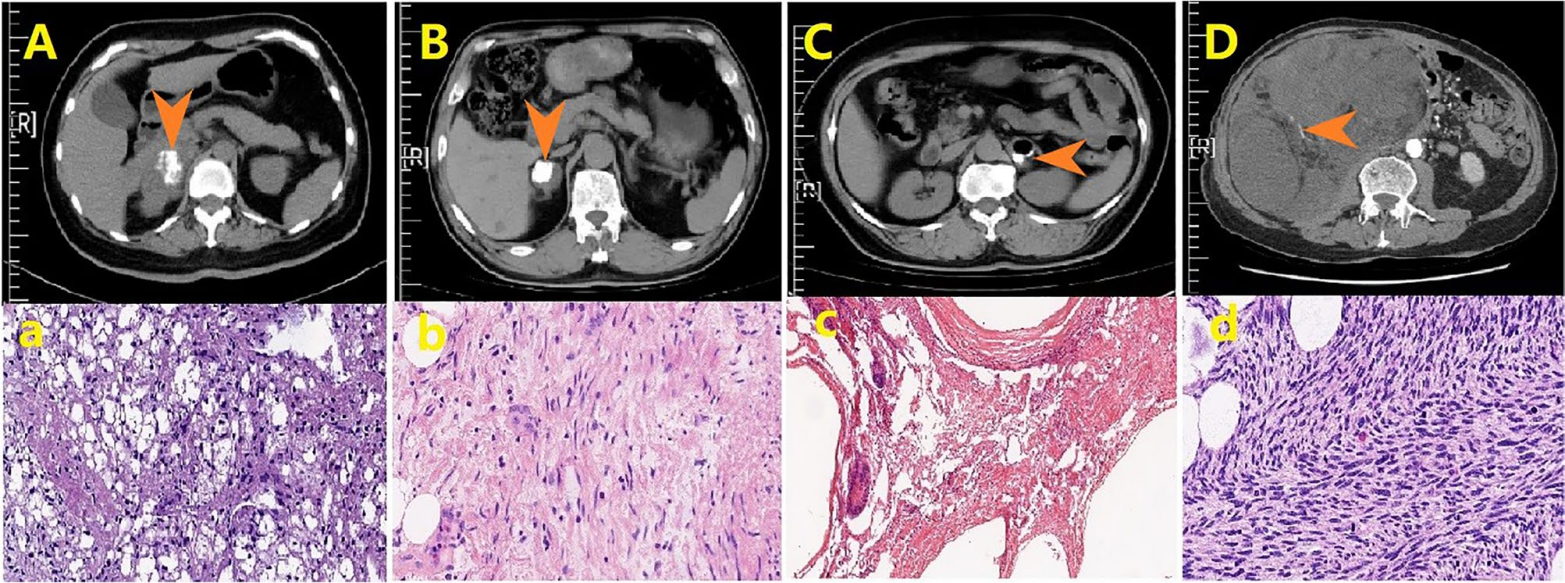
(**A**) CT of adrenal cortical carcinoma: scattered calcification inside the tumor, the tumor is lobulated with an unclear boundary. **(a)** H&E staining of adrenal cortical carcinoma, magnification × 400: most cells are dark, with a patchy density showing nuclear abnormalities, clear nucleoli and nuclear mitosis, invading blood vessels or penetrating the capsule with extensive hemorrhage and necrosis. **(B)** CT of adrenal ganglioneuroma: nodule calcification inside the tumor. **(b)** H&E staining of adrenal ganglioneuroma, magnification × 400: scattered maturely differentiated gangliocytes are seen in unmyelinated nerve fibers. **(C)** CT of mature adrenal teratoma: patchy calcification with the CT value high as 500HU. **(c)** H&E staining of mature adrenal teratoma, magnification × 100: well differentiation tissues contain 3 germ cell layers (ectoderm, mesoderm and endoderm). **(D)** CT of adrenal liposarcoma: sporadic calcification and punctate distribution inside the tumor. **(d)** H&E staining of adrenal liposarcoma, magnification × 400: adipoblasts and mature adipocytes in different differentiation stages show obvious heterogeneity.

The 75 adrenal lesions with calcification comprised 14 types of pathology (Table 2), and the representative CT and pathology image of the 8 typical sub-groups were shown in Fig. 1 and 2. The top 5 pathology were cyst in 29(38.7%) cases, myeloid lipoma in 10(13.3%) cases, cortical adenoma in 10(13.3%) cases, angiolymphangioma in 6(8.0%) cases, and schwannoma in 5(6.7%) cases. There were 4 cases of malignant tumors, including 2(2.7%) cases of adrenocortical carcinoma (ACC), one(1.3%) case of liposarcoma, and one(1.3%) case of gastric cancer metastatic to the adrenal gland.

### Survival

By April 2021, 71 were benign tumors patients had survived with a median survival period of 36.5 months (IQR = 25.5–62.5 months). Of the 4 patients with malignant tumors, the patient with liposarcoma died after a 63.2-month follow-up period (from the operation date of 09-11-2014 to 11-21-2019). The two patients with ACC were postoperatively controlled by Mitotan with no sign of recurrence or metastasis during the 82.6- and 12.2-month follow-up period respectively, and they are still under watchful observation at present. The patient with gastric adenocarcinoma metastatic to the adrenal gland is still alive after a 27.1-month follow-up period. The 4 patients with endocrine dysfunction preoperatively, including three with primary hyperaldosteronism and one with Cushing syndrome, achieved complete symptomatic remission and are still alive.

### Correlation analysis

**Figure 3:**
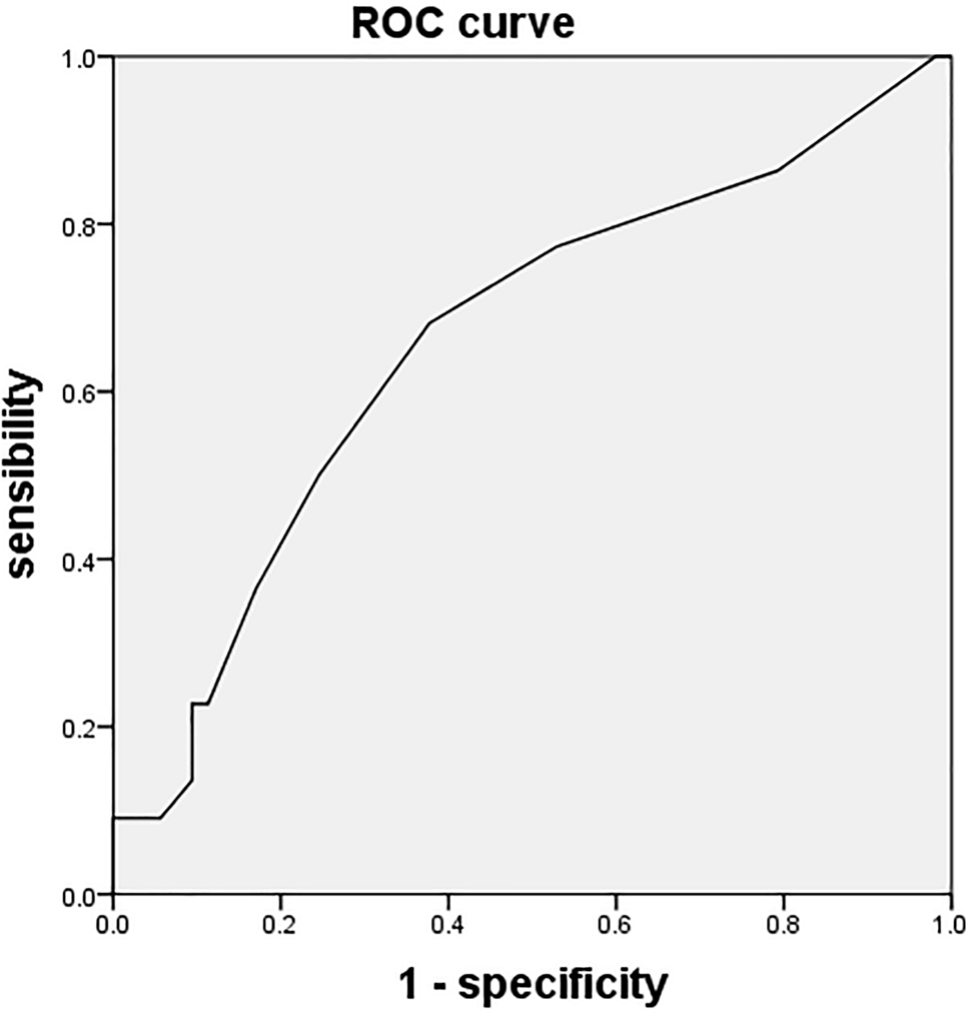
AUC of the maximum diameter ROC curve of the adrenal lesions was 0.662, suggesting that using the maximum diameter as the prediction model represents certain accuracy. The diameter, sensitivity and specificity corresponding to the maximum Youden index value were 4.5 cm, 0.682 and 0.623, respectively.

According to the definition of clinical benefit, 22 patients achieved clinical benefit, including 4 malignant tumor patients and 18 patients with improved clinical symptoms. Correlation analysis showed that clinical benefit was not correlated with calcification distribution (peripheral or internally scattered) (*P* = 0.106), calcification area ≥ 50% (*P* = 0.617), and mass enhancement (*P* = 0.720). Clinical benefit was significantly correlated with the maximum diameter of the adrenal lesion (*p* = 0.025). The median maximum diameter in benefit group *vs.* non-benefit group was 5.5 cm(IQR = 3.7–7.4 cm) *vs.* 3.7 cm(IQR = 2.8–5.4 cm). Area of under curve (AUC) of the maximum diameter receiver operating characteristic (ROC) curve of the adrenal lesions was 0.662 (Fig. 3), and the diameter, sensitivity and specificity corresponding to the maximum Youden index value were 4.5 cm, 0.682 and 0.623, respectively.

## Discussion

Calcification refers to the deposition of calcium salt in the body when the human organ or tissue undergoes necrosis under the action of certain factors. Imageologically, calcification presents as a stone-like strong echo or high-density calcareous precipitation. It can occur in various human organs and tissues, but the detection rate of calcification in the adrenal gland is relatively low^1,2^. It is reported that adrenal calcification can be secondarily attributed to internal hemorrhage and necrosis of an adrenal tumor, or primarily to calcareous diseases of the adrenal gland such as hemangioma and cyst^1,2^. Adrenal calcification can also be secondary to tuberculosis, or malignant metastasis from other systems^3–6^. There are controversies over whether surgery is necessary for such diseases at present. So we summarize our 8-year experience of adrenal lesions with calcification and discuss the rational indications for surgical treatment for the sake of avoiding unnecessary surgical intervention.

The pathological types of adrenal lesions with calcification vary with reports from authors in different periods^3–7^. In 2008, Bhargav et al.^5^ from Indian reported that calcified adrenal lesions were more likely to be seen in pheochromocytoma, adrenal cyst and myelolipoma. In 2011, Bin et al.^6^ from China reported that adrenal calcification was more likely to be seen in adrenal hemorrhage, adrenal angioma, adrenal pheochromocytoma, adrenal cortical adenocarcinoma, adrenal neuroblastoma, and adrenal TB. As the data reported in this study was obtained after 2013, the pathological types are somewhat different from those reported previously. Altogether 14 pathological types, including 11 types of benign lesions and 3 types of malignant lesion (Table 2), were identified in this study. Calcified adrenal lesions were more likely to be seen in adrenal cyst, myelolipoma, cortical adenoma, angiolymphangioma, and schwannoma (Table 2). There were also four patients with malignant tumors, including two(2.7%) with ACC, one(1.3%) with liposarcoma, and one with gastric cancer metastatic to the adrenal gland.

Calcification is seen in about 15% adrenal cysts, and usually located in the periphery of the cyst with water-like uniform density inside with no enhancement on CT^7^. Microscopically, the fibrous capsule wall tissue is covered with the pseudostratified ciliated columnar epithelium and inflammatory cell infiltration in the mesenchyma. Most adrenal cysts are hemorrhagic, with hemosiderosis and calcification contained in the cyst wall. In this study, 29(38.7%) cases of adrenal cyst were detected. Calcification in most pseudocysts was located peripherally on CT imaging. The main difference between adrenal cyst and pseudocyst is reflected in the microscopic finding that the cyst wall of the latter is made up of inflammatory granulation tissue or fibrous tissue with no coverage of epithelial cells on the cyst wall surface^3,6^.

Fat density can be seen on CT in myelolipoma and mature teratoma, and difference between them is the microscopic finding that fat lipocytes in myelolipoma are uniformly distributed, with scattered calcification inside but the CT value of calcification is not high. In contrast, calcification lesions in teratoma are mostly patchy or curved, with the CT value high as 500HU^1,7^. Adrenocortical adenoma presents as a round or oval nodule containing blood-supply on CT, and calcification is scattered and can be seen in the periphery or inside^7^. Microscopically, adrenocortical adenoma is composed of clear and compact cells; the tumor cells are arranged in a cord-like or acinar manner, with mitosis rarely seen. In this study, 10 cases of adrenocortical adenoma were found.

It is reported that calcification occurs in about two-thirds of adrenal angiolymphangioma^8^. This study included 6 cases of adrenal angiolymphangioma. Calcification located in the periphery and presented a patchy shape on CT. In some cases, partitioned calcification foci can be seen inside. Histologically, the cystic spaces were lined by a single layer of flattened or focally hobnailed cells without atypia resembling that of normal lymphatics. In the wall of several larger cystic spaces fascicles of smooth muscle are visible^8^.

This study included 5 cases of schwannoma. Calcification in schwannoma mainly located in the periphery on CT, forming an “egg shell-like” shape encircling the tumor, with an CT value of calcification between 200 and 300HU^7^. Microscopically, the tumor cells are spindle-shaped with no clear boundaries between cells, and cells are arranged in a whirlpool or palisade manner. Necrotic change can be seen in most pheochromocytomas, with a small number of chromaffin cells accompanied with calcification^1^. This study only included one case of pheochromocytoma, presenting a ring-like shape on CT, with a CT value of calcification larger than 500HU. Microscopically, most tumor cells are polygonal with rich granular or vacuolar alkalophilic cytoplasm and clear nucleoli, and cells are arranged in a nest-like, trabecular or acinar shape. Immunohistochemistry mainly shows strongly positive Chromogranin A (CgA). Calcification in ganglioneuroma presents as nodules inside the tumor on CT. The calcification area in one case of the present study exceeded 50%. Gross examination showed a capsule around the tumor, and the cross section looked off-white and hard in consistency. Light microscopically, patchy or scattered maturely differentiated gangliocytes are seen in unmyelinated nerve fibers.

Calcification in ACC is spotty on CT image, scattering inside the tumor with a relatively low CT value of calcification (< 100Hu)^9^. The maximum diameter is relatively large, and the tumor is lobulated with an unclear boundary. The maximum diameter in our two cases of ACC is larger than 7 cm. Microscopically, most cells are dark, with a patchy or focal density showing nuclear abnormalities, clear nucleoli and nuclear mitosis, invading blood vessels or penetrating the capsule with extensive hemorrhage and necrosis. This study included one case of liposarcoma with 20 cm in diameter. Calcification presents as a sporadic and punctate distribution inside the tumor on CT, with value of calcification less than 100 HU. Microscopically, adipoblasts and mature adipocytes in different differentiation stages show obvious heterogeneity. This study included one case of gastric adenocarcinoma metastatic to the adrenal gland. Calcification presents a scattered spotty distribution on CT, with a CT value of calcification lower than 100 HU. Microscopically, there is little lipid, and typical gastric adenocarcinoma cells can be seen.

Additionally, calcification of adrenal TB mostly appears in the middle and late stages of the tuberculosis, affecting both sides in about 91% cases^10^, often accompanied with manifestations of TB in other organs. At present, it is generally accepted that anti-TB drug therapy is the mainstay of treatment for adrenal TB, with no necessity of surgery. For this reason, we did not perform surgery in a single case of adrenal TB in our series during the past 8-year period. Lau et al. reported that adrenal calcifying fibrous tumor (CFT) has the following pathological characteristics, including diffuse positive immunoreactivity for factor XIIIa and absence of reactivity for muscle specific actin, smooth muscle actin, and anaplastic lymphoma kinase^11^. We did not find a single case of CFT in our study, probably due to regional factors.

Till now, there are still controversies over whether surgery is necessary for adrenal lesions with calcification^4–6^. For patients with adrenal lesions < 3 cm without obvious enhancement and without endocrine dysfunction, active watching is recommended. In 2011, Bin et al. reported that patients with adrenal tumors between 4 and 6 cm could be observed, and surgical intervention is necessary for patients with tumors ≥ 6 cm because of the increased probability of malignancy^6^. In this study, we set clinical benefit as the criterion of surgical indication selection. Of the 75 patients involved, 22 patients achieved surgical benefit and the remaining 53 patients failed to achieve surgical benefits, with a benefit rate of 29.3%. Influencing factors analysis revealed that maximum diameter of the adrenal lesion was significantly correlated with surgical benefit, with a median value of 5.5 cm (IQR = 3.7–7.4 cm) *vs.* 3.7 cm (IQR = 2.8–5.4 cm) (*p* = 0.025). AUC of the maximum diameter ROC curve of the adrenal lesions was 0.662, suggesting that using the maximum diameter as the prediction model represents certain accuracy. The diameter, sensitivity and specificity corresponding to the maximum Youden value was 4.5 cm, 0.682 and 0.623 respectively. Besides whether 4.5 cm could be used as the cutoff point of surgical selection needs to be considered in the context of the symptoms such as hypertension, and whether there is possibility of malignancy. The mechanism underlying postoperative improvement of symptoms such as hypertension and low back pain may be related to the relief of psychological stress and/or tumor compression on the normal adrenal tissue or surrounding visceral organs^12,13^, and further study is still required.

The surgical approach should be selected after a comprehensive consideration of the tumor size and location, endocrine function and the surgeon’s preference. According to our experience and practice, retroperitoneoscopy is preferable for tumors < 8 cm, and transperitoneal laparoscopy for tumors ≥ 8 cm. For giant or suspected malignant tumors, transperitoneal open surgery is recommended. In this study, we performed retroperitoneoscopy in 39(52%) cases and transperitoneal laparoscopy in 27(36%) cases. Transperitoneal open surgery were performed in 9(12%) cases, and the median value of maximum diameter of the adrenal lesion was 11.8 cm(IQR = 7.5–13.7 cm).

Whether the adrenal gland should be preserved still remains controversial. Generally, preoperative CT is required to assess the morphology of the bilateral adrenal glands and the overall adrenal function. If there is a clear boundary between the tumor and the adrenal gland and the adrenal central vein can be preserved completely, preservation of the adrenal gland can be considered provided en block R0 resection of the tumor is ensured. Once malignancy is suspected, the adrenal gland should be removed completely to ensure the tumor-free criterion and avoid postoperative local implantation and infection due to tumor rupture or leakage of inflammatory and necrotic substances. Of the 75 cases in our series, total resection of the adrenal gland and lesion was performed in 38(51%) cases including four cases of malignancy. Part of the normal adrenal gland was preserved in the remaining 37(49%) cases to ensure normal secretion of adrenal endocrine hormone after operation.

Our study has some limitations due to retrospective design, single center and tertiary care patient population that might affect generalizability to some degree. In addition, the number of patients with adrenal calcified lesion was somewhat limited, though our hospital is a high-volume center in adrenal disease in China. Furthermore, magnetic resonance imaging should be widely applied in this entity in the future. As outlined above, longitudinal follow-up will be required to assess the optimal surgery indication for adrenal lesions with calcification.

## Methods

### Statement

(1) All experiments, including methods and operations were approved by the Ethics Committee of Ruijin hospital, Shanghai Jiao Tong University School of Medicine, China. (2) All experimental methods were performed in accordance with guidelines and regulations of the Ethics Committee of Ruijin hospital, Shanghai Jiao Tong University School of Medicine, China. Informed consent was obtained from all patients and also from legal authorized representative of dead patient.

### Patient population

We reviewed 5057 patients with adrenal lesions who received surgery at our institution from January 2013 to April 2021. Patients were excluded for lack of pathology, preoperative CT, or laboratory finds. Finally 75 patients with adrenal calcification concurrence of both preoperative CT and postoperative pathology were included in this study. The clinical presentations, laboratory tests, imaging findings, surgical records and follow-up data of the 75 cases of adrenal lesions with calcification were collected by the electronic case system. The location and endocrinological diagnosis of the lesions were determined preoperatively by contrast-enhanced CT scan and endocrine function tests respectively. Tuberculosis (TB) was excluded preoperatively by chest X-ray, purified protein derivative skin test and detection of Mycobacterium tuberculosis from urine.

### Surgical intervention and definition of clinical benefit

The surgical modality (retroperitoneoscopy, transperitoneal laparoscopy or open surgery adrenectomy) was decided by the attending surgeons with more than 10 year’s experience, depending on the location and size of the calcified adrenal lesions. The pathology reports were finally reviewed by the full-senior pathologists with more than 20 year’s experience. Clinical benefit was defined as relief of symptoms or/and removal of the malignant tumor.

### Statistical analysis

Continuous variables were expressed as median and interquartile range (IQR) and compared using Student t test for normally distributed data and Mann–Whitney U test for non-normally distributed data. Categorical variables were compared using the chi-square and Fisher’s exact tests. Association between clinical benefit and the parameters was analyzed in a multivariable regression model. All *p* values were two-tail and *p* < 0.05 was considered statistically significant. Data were analyzed using SPSS version 20.0 (SPSS Inc., Chicago, IL, USA).

## Conclusions

Most adrenal lesions with calcification are pathologically benign. The clinical benefit is significantly correlated with the maximum diameter of the lesion, and 4.5 cm may be considered as the cutoff point of surgical selection. The exact mechanism underlying the clinical improvement needs to be further explored. Strict control of the surgical indication can avoid unnecessary surgical intervention.

## Ethical statement

All experimental procedures were approved by the Ethics Committee of Ruijin Hospital, Shanghai Jiao Tong University School of Medicine, China. This retrospective chart review study involving human participants was in accordance with the ethical standards of the institutional and national research committee and with the 1964 Helsinki Declaration and its later amendments or comparable ethical standards. All patients were informed thoroughly and informed consent was obtained from all patients and also from Legal authorized representative of dead patient.

## Data Availability

The datasets used and/or analysed during the current study are available from the corresponding author on reasonable request.

## Abbreviations

ACC: Adrenal cortical carcinoma
AUC: Area of under curve
CFT: Calcifying fibrous tumor
CgA: Chromogranin A
CT: Computed tomography
IQR: Interquatile range
ROC: Receiver operating characteristic
TB: Tuberculosis
